# Characterization of *Escherichia coli* harboring colibactin genes (*clb*) isolated from beef production and processing systems

**DOI:** 10.1038/s41598-022-09274-x

**Published:** 2022-03-29

**Authors:** Manita Guragain, John W. Schmidt, Norasak Kalchayanand, Aaron M. Dickey, Joseph M. Bosilevac

**Affiliations:** Meat Safety and Quality Research Unit, US Department of Agriculture, Agricultural Research Service, U.S. Meat Animal Research Center, State Spur 18D, P.O. Box 166, Clay Center, Nebraska 68933 USA

**Keywords:** Food microbiology, Bacterial genetics, Colorectal cancer

## Abstract

Certain strains of *Escherichia coli* possess and express the toxin colibactin (Clb) which induces host mutations identical to the signature mutations of colorectal cancer (CRC) that lead to tumorigenic lesions. Since cattle are a known reservoir of several *Enterobacteriaceae* including *E. coli*, this study screened for *clb* amongst *E. coli* isolated from colons of cattle-at-harvest (entering beef processing facility; n = 1430), across the beef processing continuum (feedlot to finished subprimal beef; n = 232), and in ground beef (n = 1074). Results demonstrated that *clb*^+^ *E. coli* were present in cattle and beef. Prevalence of *clb*^+^ *E. coli* from colonic contents of cattle and ground beef was 18.3% and 5.5%, respectively. *clb*^+^ *E. coli* were found susceptible to commonly used meat processing interventions. Whole genome sequencing of 54 bovine and beef *clb*^+^ isolates showed *clb* occurred in diverse genetic backgrounds, most frequently in phylogroup B1 (70.4%), MLST 1079 (42.6%), and serogroup O49 (40.7%).

## Introduction

Colorectal cancer (CRC), the fourth most common cancer in the United States^1^, is a multifactorial disease associated with risk factors such as obesity, genetics, and lifestyle including smoking, alcohol, and diet (as reviewed in^2^). The International Agency for Research on Cancer reports a positive association between high consumption of red meat and CRC, thereby, classifying red meat as “probably carcinogenic to humans”. Association between CRC and red meat consumption is classically attributed to the formation of carcinogenic compounds generated during high temperature cooking^3^. However, as reviewed in^4^, intrinsic properties of red meat itself do not provide adequate mechanistic links between increased CRC risk and red meat consumption. This warrants investigation into extrinsic factors such as meat-borne tumorigenic bacteria as a missing link between red meat and CRC.

The abundance of *E. coli* carrying DNA damaging genes like colibactin (*clb*) and cytotoxic necrotizing factor (*cnf)* is increased in colon biopsies from CRC patients compared to non-CRC patients and healthy individuals^5^. Clb is an important virulence factor of *E. coli* and other *Enterobactericeae*^6,7^. This genotoxic, nonribosomal, hybrid peptide-polyketide is encoded within the 54 kb polyketide synthase (*pks*) genomic island^8^. Clb expressed by *clb*^+^ *E. coli* alkylates DNA and induces double strand DNA breaks and interstrand cross linking of mammalian DNA^8–10^. These cross-links result in chromosomal aberrations, cell cycle arrest, and cell senescence^8,11^. Mouse model studies show Clb induces the formation of invasive colonic tumors associated with increased DNA damage^12^. Unique single strand breaks and indels induced by Clb are found to be identical with mutational signatures from a subset of human cancer genomes, particularly, colorectal cancer (CRC) and though rare, in tumors derived from head, neck, urinary tract^13^, and oral squamous carcinoma^14^.

The gastrointestinal tract of cattle is a natural reservoir of both commensal and pathogenic *E. coli*. These *E. coli* are categorized using various schemes which allow quick assignment of an isolate to specific host, pathogenesis, and phylogenetic lineages. *E. coli* are defined based on the variation in O-antigen of outer membrane lipopolysaccharide to any of 185 O-serogroups^15^. DNA sequence variations in internal regions of multiple housekeeping genes define clonal diversity and categorize *E. coli* into different multilocus sequence type (MLST) profiles^16^. Lastly, there are 8 phylogenetic groups of *E. coli* defined by sets of five genes, each set specific to individual phylogroup^17^. During beef processing, *E. coli* of these various sorts from hides and feces may directly contaminate the carcasses that are intended to be further processed into final beef products ^18,19^. Despite the advances in pathogen control in beef production and processing, ground beef still continues to be an important source of *E. coli* infections that occasionally lead to recalls and outbreaks^20^ (as reviewed in^21^). Therefore, the beef chain (cattle to finished beef) was examined for the presence of *clb*^+^ *E. coli* to test the hypothesis that these *E. coli* may be contributing to the classification of beef as a potential carcinogen.

The results presented herein show the potential of cattle as a reservoir and beef as a vehicle of *clb*^+^ *E. coli*. Arbitrarily isolated *E. coli* from cattle and ground beef were found to carry *clb* in their genomes. Further, currently used processing interventions are shown to be effective control measures that can reduce *clb*^+^ *E. coli* contamination of beef. Whole genome sequencing showed that the organization of the *pks* island is highly conserved in *clb*^+^ *E. coli* isolated from cattle and beef, and revealed that the majority of *clb*^+^ isolates from cattle and beef belong to a limited number of phylogroups, serogroups, and MLST types.

## Results

### Prevalence of ***clb***^+^***E. coli*** in US beef cattle

**Figure 1:**
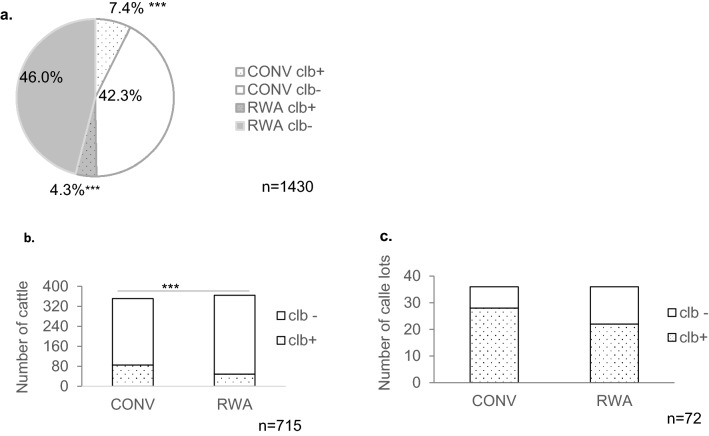
*clb*^+^ *E. coli* among cattle-at-harvest. (**a**) Percentage prevalence of *clb*^+^ *E. coli* among all generic *E. coli* isolated from cattle (**b**) Number of animals carrying *clb*^+^ *E. coli*. (**c**) Number of cattle lots carrying *clb*^+^ *E. coli.* Statistical significance was calculated by Fisher’s exact test. *, significant (*p* < 0.05); ***, extremely significant (*p* < 0.001). CONV, conventionally raised; RWA, Raised without antibiotics.

*E. coli* isolates (n = 1430) previously collected from colons of 709 eviscerated cattle ^22^ were screened in order to determine the prevalence of *clb*^+^ *E. coli* in beef cattle. Overall, 18.7% (134/715) of cattle carried *E. coli* with *clb* in their genomes. On a per animal basis a significantly higher (*P* < 0.05) number of conventionally raised (CONV) cattle (85/351) carried *clb*^+^ *E. coli* in their colons compared to raised without antibiotics (RWA) cattle (49/364) (Fig. 1). Since two isolates were selected per animal^22^, this amounted to 11.7% (167/1430) of the cattle borne *E. coli* testing positive for *clb.* Significantly higher prevalence of *clb*^+^ *E. coli* was observed among isolates from CONV cattle (15% of CONV isolates) compared to RWA cattle (8.5% of RWA isolates) (Fig. 1). However, this difference between the production system was not reflected in the cattle lots, with the number of CONV (28/36) and RWA cattle lots (22/36) carrying *clb*^+^ *E. coli* not being significantly different (*P* > 0.05) (Fig. 1). *clb*^+^ *E. coli* were isolated from cattle during all seasons (Winter, 11.7%, Summer, 9.2%, and Fall, 8.2%), with highest prevalence during spring season (17.5%, p < 0.05) (Supplementary Fig. S1).

### Prevalence of *clb*^+^*E. coli* across beef processing continuum

Bacteria introduced during early stages of meat processing may get carried over across the production continuum, ultimately reaching the final products. To identify the distribution of *clb*^+^ *E. coli* across the beef production continuum, 232 generic *E. coli* collected from three lots of conventionally raised fed beef cattle harvested at the same beef processing plant^23^ were utilized. The isolates were collected at seven different stages of the processing continuum from live animals to finished products. Results indicated that prevalence of *clb*^+^ *E. coli* was extremely low across the beef processing continuum. *clb*^+^ *E. coli* were found only amongst isolates from the earliest points in the continuum: feedlot fecal (1/36) and harvest hide sponge (1/36) from two different lots (Supplementary Table T1).

### Prevalence of *clb*^+^*E. coli* in retail ground beef

**Figure 2:**
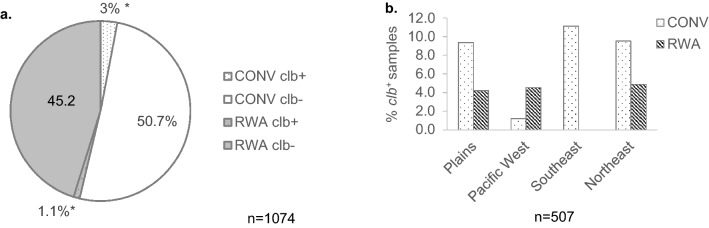
*clb*^+^ *E. coli* occurring in ground beef. (**a**) Percentage prevalence of *clb*^+^ *E. coli* in ground beef among all generic *E. coli* isolated; (**b**) Percentage of ground beef samples carrying *clb*^+^ *E. coli.* Statistical significance was calculated by Fisher’s exact test. *, significant (*p* < 0.05). CONV: ground beef produced from conventionally raised cattle; RWA: ground beef produced with label claim of “from beef raised without antibiotics”.

To estimate the prevalence of *clb*^+^ *E. coli* in ground beef, 1,074 generic *E. coli* previously isolated from 507 retail ground beef samples collected from 6 US cities^24^ were screened. Overall, prevalence of *clb*^+^ *E. coli* in ground beef samples was found to be 5.5% (28/507) (Fig. 2). With two isolates selected per sample^24^, 4.1% (44/1074) of the generic *E. coli* from ground beef carried *clb* in their genome. *clb*^+^ *E. coli* appeared significantly lower (*P* < 0.05) among isolates recovered from ground beef with RWA label claims (2.4%) compared to isolates from CONV ground beef (5.5%). However, there was no significant difference on a per sample basis where *clb*^+^ *E. coli* were isolated from 9 of 240 RWA and 19 of 267 CONV samples. Interestingly, among the samples from the southwestern region, *clb*^+^ *E. coli* were entirely absent from RWA samples (Fig. 2). Prevalence of *clb* appears to be lowest among isolates from ground beef purchased in the Pacific West region (1.9%) compared to Plains (5.3%), Southeast (4.6%), and North East (5.5%) regions (Supplementary Fig. S2).

### Susceptibility of *clb*^+^*E. coli* towards meat processing interventions

Forty-four selected *clb*^+^ *E. coli were* screened for susceptibility towards current beef processing antimicrobial compounds used as interventions by using a benchtop killing assay. *clb*^+^ *E. coli* were treated with 2% lactic acid (LA), 200 ppm Peroxyacetic acid (PAA), 300 ppm Bromine (BR), and hot water (HW, 80 °C) in a 96-well setting. Fold change in cell density (OD600) of treated samples compared to untreated samples was calculated and compared to that of *E. coli* O157:H7 FSIS-4. It was observed that *clb*^+^ *E. coli* were susceptible to the antimicrobials with fold reductions for hot water, 2.5 ± 0.4 to 4.2 ± 2.2; LA, 2.5 ± 0.7 to 3.9 ± 1.0; PAA, 2.6 ± 0.4 to 4.7 ± 2.3; and BR, 2.0 ± 1.2 to 5.4 ± 2.8. All strains were as sensitive or more sensitive to the treatments as the reference *E. coli* O157:H7 FSIS-4 isolate^25^ (Supplementary Fig. S3).

**Figure 3:**
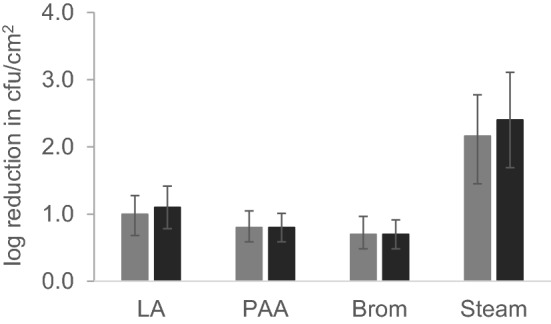
Beef flank inoculation assay. LA, 2% lactic acid (1 min). PAA, 200 ppm peroxyacetic acid (1 min). Brom, 300 ppm BoviBromine (1 min). Steam, flank surface temperature achieved 82 °C with dry steam (15 s). Statistical significance between *clb*^+^ *E. coli* and reference *E. coli* O157:H7 was calculated by Fisher’s exact test (*p* ≥ 0.05, not significant). Data presented represents average of 2 biological replicates, 12 technical replicates each on individual beef flanks. *clb*^+^ *E. coli*, * E. coli* O157:H7.

Next, to assess the efficacy of antimicrobial interventions on controlling *clb*^+^ *E. coli* on beef carcass surfaces, an inoculum pool of 4 *clb*^+^ isolates and the reference *E. coli* O157:H7 FSIS-4 isolate was inoculated on beef flank (cutaneous trunci muscle) surfaces and treated in the USMARC pilot processing plant. The inoculated flanks were exposed to antimicrobial interventions and log reductions in Colony forming unit (CFU) per cm^2^ were calculated. *clb*^+^ isolates inoculated on the beef flank surfaces were found to be equally susceptible to LA, PAA, and BR with Log reductions of 0.7–1.3, 0.6–1.0, and 0.4 ± 1.0 CFU/cm^2^ respectively. Dry steam was found to be most effective at reducing *clb*^+^ *E. coli* on beef flank surfaces (1.6–2.8 2 Log CFU/cm^2^reduction) (Fig. 3). There were no statistically significant differences between *clb*^+^ *E. coli* and the reference *E. coli* O157:H7 FSIS-4 (log reduction: steam, 1.7–3.1; LA, 0.8–1.4 1; PAA, 0.6–1.0; BR, 0.5–0.9) (*P* > 0.05, one-way ANOVA).

### Genome characteristics of *clb*^+^*E. coli*

An in-house genome analysis pipeline was used to assemble the genomes, screen them against Center for Genomic Epidemiology (CGE) databases and obtain the assembly statistics. Assembly statistics and characteristics of fifty-four *clb*^+^ *E. coli* genomes are summarized in Supplementary Data File SD1.

**Figure 4:**
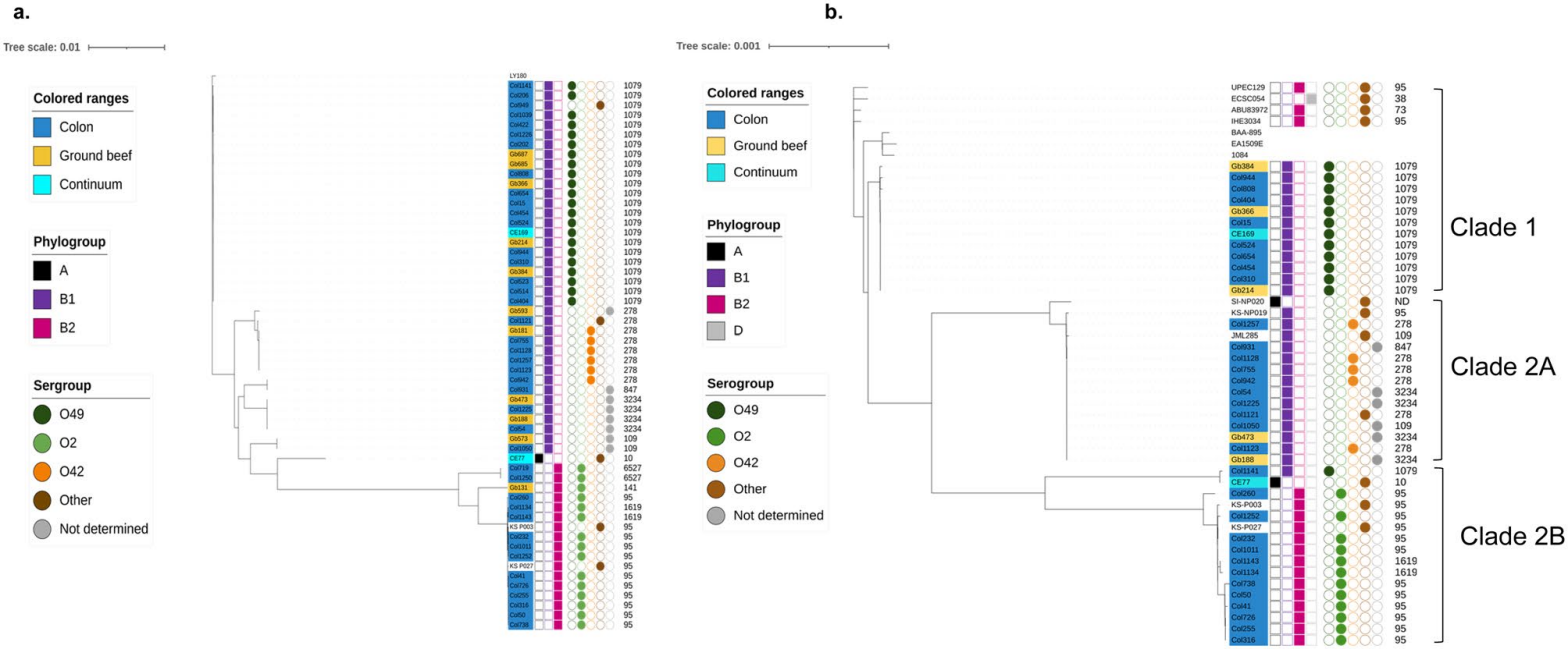
Maximum likelihood phylogenetic tree of (**a**) 54 core genomes of *clb*^+^ *E. coli* isolated from beef production and processing continuum, and (**b**) 39 *pks* island sequences extracted from the contigs carrying full length *pks* island (*clbA*-*clbS*). Phylogenetic trees were constructed using IQ-tree and visualized with iTOL v6. Numbers on the outermost column represent multilocus sequence types (MLST).

Phylogroups of *clb*^+^ *E. coli* were determined *in-silico* with ClermonTyping 21.03^26^. Overall, 70.4% (38/54) of the *clb*^+^ *E. coli* in this study belong to phylogroup B1, followed by B2 (27.8%, 15/54), and A (1.9%, 1/54) (Fig. 4). Among *clb*^+^ *E. coli* isolates from the colons of cattle, prevalence of phylogroups B1 and B2 were 65.9% and 34.1% respectively. Among ground beef *clb*^+^ *E. coli*, 90.9% belonged to phylogroup B1, while 9.1% to B2. One *clb* + isolate from beef processing continuum belonged to phylogroup A, and the other to phylogroup B1. Owing to difference in number of genomes, no attempt was made to compare phylogroups between the sample sources.

Based on *in-silico* O-serotyping using SerotypeFinder version 2018_09_24^27^, the most pre-dominant O-serogroup among sequenced *clb*^+^ isolates was O49 (40.7%, n = 22), followed by O2 (27.8%, n = 15), and O42 (11.1%, n = 6), and other O-groups (7.4%, n = 4). O-serogroup could not be determined for 7 isolates (Fig. 4). *In-silico* multilocus sequence typing using MLST version 2019_05_08^28^ showed three-quarters of the sequenced *clb*^+^ *E. coli* in our study belonged to three MLST types; 23 to MLST 1079, 10 to MLST 95, and 8 to MLST 278 (Fig. 4).

**Figure 5:**
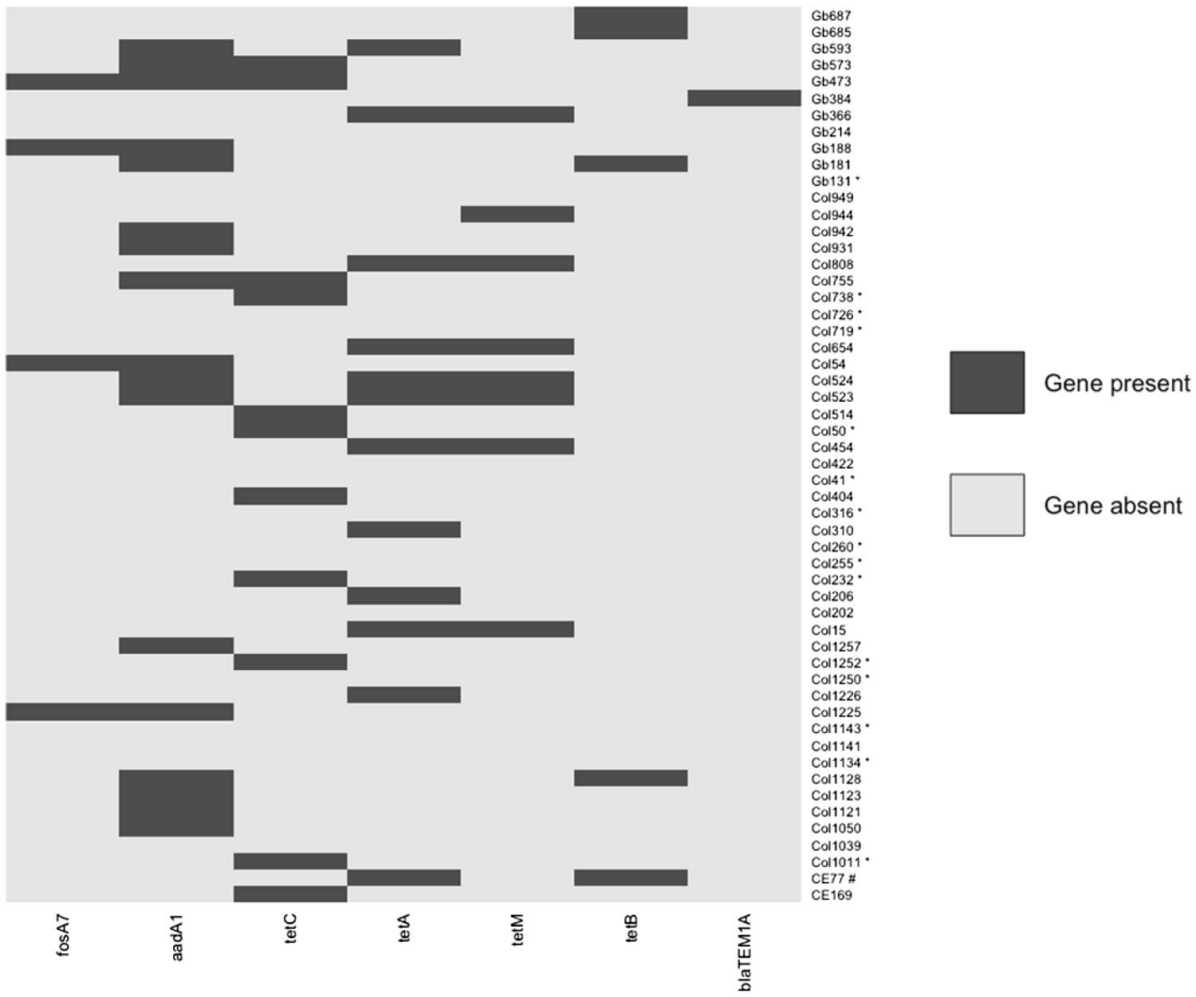
Distribution of acquired antibiotic resistance genes in *clb*^+^ *E. coli*. Heat map was created and visualized using ggplot2 package of R. Column represents *E. coli* isolates and row represents acquired antibiotic resistance genes. *, Phylogroup B2; # Phylogroup A.

Antibiotic resistance genes were identified by ResFinder version 2020_02_06^29–31^. Prevalence of genes associated with tetracycline resistance (*tetA*, *tetB*, *tetC*, *tetM*), aminoglycoside resistance (*aadA1*), fosfomycin resistance (*fosA7*), and beta-lactamase resistance (*bla TEM-1A*) were 51.8, 31.5, 7.4, and 1.9% respectively (Fig. 5) (Supplementary Data File SD1).

**Figure 6:**
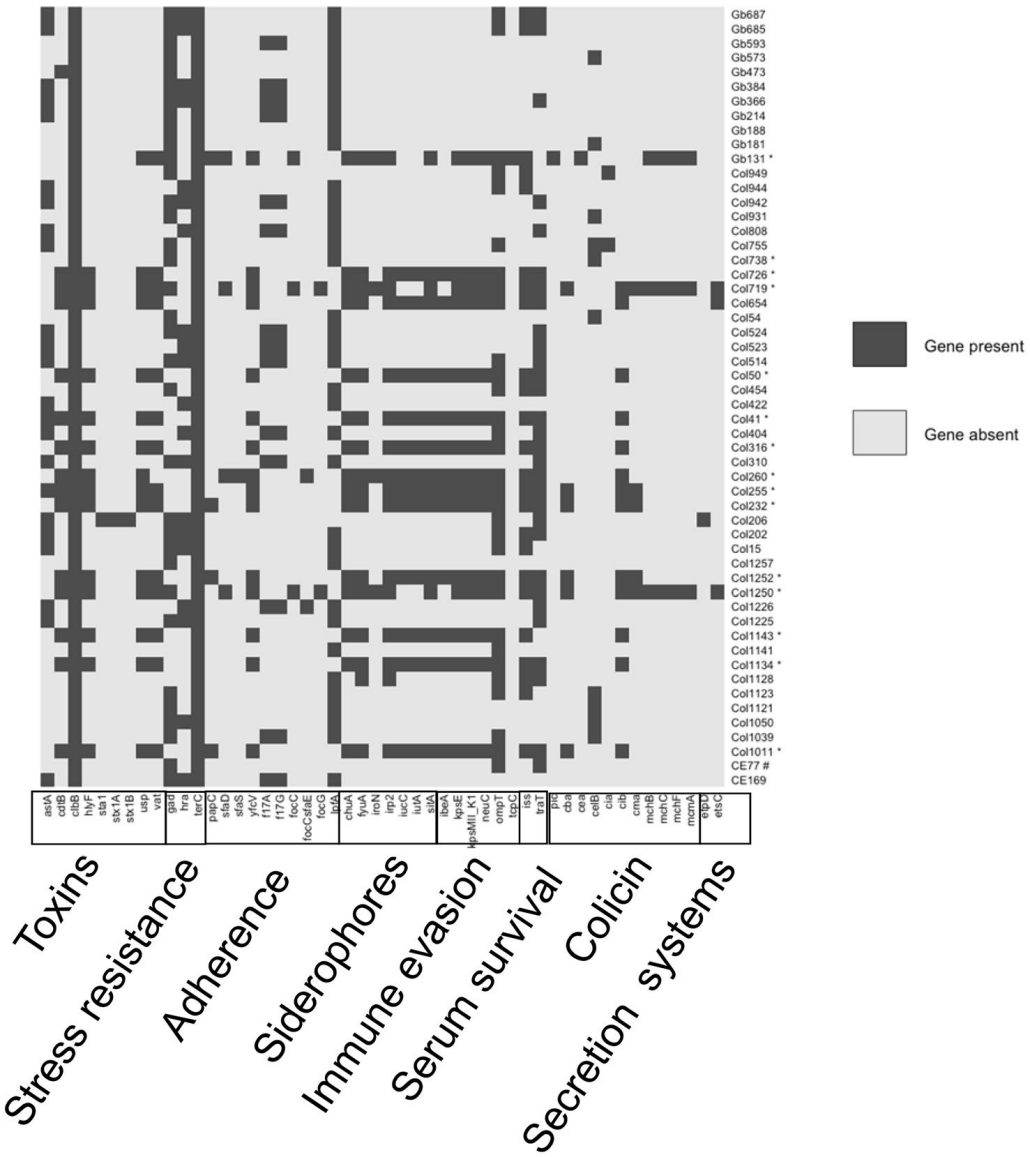
Distribution of acquired virulence genes in *clb*^+^ *E. coli*. Heat map was created and visualized using ggplot2 package of R. Column represents *E. coli* isolates and row represents acquired virulence genes. *, Phylogroup B2; # Phylogroup A.

VirulenceFinder 2.0^32,33^ identified 50 virulence genes belonging to several functional categories like toxin, stress resistance, adherence, siderophores, immune evasion, serum survival, colicin, and secretion system, in a decreasing order of abundance among *clb*^+^ isolates. Tellurium resistance gene *terC*, with implicated role in general stress tolerance in the host environment, was present in all *clb*^+^ isolates identified in this study. Other highly distributed genes were long polar fimbriae *lpfA* (67%), glutamate decarboxylase *gad* (63%), outer membrane protease *ompT* (57%), and outer membrane protein complement resistance *traT* (57%). Cytolethal distending toxin *cdtB*, another genotoxin which promotes colorectal cancer^34^, was present in 15 genomes (14 group B2 and 1 group B1). Overall, group B2 *clb*^+^ *E. coli* isolates harbored more virulence factors compared to group B1 isolates (Fig. 6) (Supplementary Data File SD1).

### Phylogenetic analyses

Core genomes of the 54 sequenced *clb*^+^ isolates were aligned with PARSNP version 1.2^35^, maximum likelihood tree was constructed with iQ-tree 1.6.10^36^ and was visualized with iTol V6^37^. The genomes distinctly grouped according to phylogroups, serogroups, and MLST profiles. Isolates from different sources as wells as different production systems (i.e., RWA and CONV) grouped together in the same clades (Fig. 4).

High sensitivity mapping using Geneious mapper in Geneious prime 2020.1.2 (Biomatters) against the reference *pks* sequence regions spanning *clbA-clbS* identified *pks* islands in all fifty-four newly sequenced genomes. Among these newly identified *pks* islands, thirty eight *pks* sequences present in full length in individual contigs were then aligned (Supplementary Fig. S4) and a maximum likelihood tree was constructed. Compared to the chromosome, G + C contents of all *pks* islands were higher (53.5% VS ~ 50.1%). Nucleotide sequence comparison showed that *pks* islands were highly conserved (> 99.64%) in comparison to each other and the reference sequences and maintained genetic synteny (Supplementary Fig. S4). When differences in nucleotide sequences were found, they mostly resulted from substitutions within genes.

Phylogenetic analysis showed that *pks* islands grouped into two major clades, largely by phylogroups, O-serogroups, and MLST profiles of the isolate in which they were found (Fig. 4). Nucleotide sequence identity between the clades ranged from 99.63 to 99.82%. Clades were not specific to sample sources or production system. Clade 1 exclusively consisted of twelve *pks* sequences belonging to group B1 isolates, all of which belonged to MLST 1079 and serogroup O49. Only one sequence from a MLST 1079 isolate grouped with clade 2 sequences. Clade 1 sequences were 100.0% identical to each other, while 99.95–99.98% identical with clusters of reference sequences from human clinical isolates. Interestingly, *pks* sequences from cattle B2 strains are phylogenetically closer to B1 strains than to the B2 human clinical reference strains. *pks* sequences in clade 2 further distinctly grouped in two subclades. Clade 2A *pks* sequences originated from twelve isolates, exclusively of phylogroup B1 and includes *pks* sequence from human commensal strain JML285. Isolates in this clade appear to be the most diverse of all three clades in context of their serogroups and MLST profile yet 100.0% identical regarding *pks* sequences, except for reference sequence SI-NP020 from a bovine isolate, which shared 99.97% identity with rest of the clade. Clade 2B consisted of fourteen sequences, of which twelve belong to isolates of phylogroup B2 and serogroup O2, sharing > 99.97% nucleotide sequence identity. Ten of these sequences belonged to MLST 95 and the remaining two MLST 1619. Two reference *pks* sequences from bovine fecal isolates KS-P003 and KS-P027 (phylogroup B2, MLST95) also clustered together with these ten sequences. The remaining clade 2B sequences belonged to isolates of group A and group B1 each and shared 99.70% identity with rest of the clade 2B.

Since clonal complex 95 (CC95) strains are known as significant extraintestinal pathogens in humans^17^, we further analyzed these strains to understand the distribution of *pks* islands. Core genomes of all *pks*^+^ CC95 strains, including two previously reported bovine strains KS-P003 and KS-P027 closely clustered together. All CC95 strains identified in this study belonged to the same serogroup (O2) and phylogroup (B2), but different serogroup than KS-P003 and KS-P027 (Fig. 4A). *pks* islands in these strains grouped together in same clade (2B), but away from *pks* islands in CC95 human isolates in clade 1 (Fig. 4B). An in-silico search for genes and primer sequences specific to unique subgroups^38^ among the CC95 *pks*^+^ strains from this study was unsuccessful therefore *fimH* typing was performed. In combination with phylogroups, *fimH typing* has been described as a useful approach in classifying strains of *E. coli*^39^. The *fimH* allele in these strains were identified using fimtype 1.0 available from center for genomic epidemiology. All CC95 strains including KS-P003 and KS-P027 were found to carry the same *fimH* allele (*fimH*15).

## Discussion

Beef-borne pathogenic *E. coli* outbreaks are often associated with diarrheagenic pathotypes, that cause acute intestinal diseases, but other beef-borne chronic infections due to non-classical pathotypic *E. coli* are often overlooked. The association of red meat consumption with increased risk of CRC^3^, which in turn is associated with increased tumorigenic bacterial populations^5,40^, undoubtedly demands the investigation of beef as a vehicle for such bacteria including *clb*^+^ *E. coli*. The *clb*^+^ *E. coli* further poses a multigenerational threat as vertical transmission has been reported in humans^41^ and experimental animals^42^, where they produce long-lasting effects. This study demonstrates that the *clb* gene cluster is present in commensal *E. coli* isolated from cattle and beef. In the U.S., cattle presented for harvest are found to be colonized by pathogenic *E. coli* (*E. coli* O157:H7 and non-O157 Shiga toxin producing *E. coli*; STEC) and *Salmonella* at rates of 1.9–34.3%^19^ and 1.7–9.2%^22,43^, respectively, using culture methods that enrich and select for the pathogen. In contrast, 18.5% of cattle were determined to carry arbitrary *clb*^+^ *E. coli* that were isolated and identified without enrichment or selective pressure. Although antimicrobial interventions focus on controlling *E. coli* O157:H7 and reduce their prevalence in finished beef products to less than a fraction of one percent^44^, arbitrary *clb*^+^ *E. coli* was found at a rate of 4.1% in finished ground beef. This is despite the fact that *clb*^+^ *E. coli* reacted equally or were more sensitive to the processing interventions tested as *E. coli* O157:H7. Thus, finished beef is a potential vehicle for *clb*^+^ *E. coli* transmission.

*E. coli* contamination of beef primarily occurs during steps of sanitary dressing when the hide is removed from the carcass, with minor inputs from the steps of evisceration^45^.Further investigation of alternate foodborne sources of *clb*^+^ *E. coli* are needed before proper risk assessments can be performed to determine the actual impact of beef-borne *clb*^+^ *E. coli* on public health. Our examination of the *E. coli* isolates recovered from the beef processing continuum were too limited in number to reveal an alternate route of *clb*^+^ *E. coli* contamination of beef. Pathogenic *E. coli*, however, have been reported to be present in beef lymph nodes, which upon grinding may release the bacteria into the ground beef^46^. Alternately there may be environmental niches where *clb*^+^ *E. coli* may persist in the processing plant environment protected within a biofilm^47^.

The overall prevalence of *clb*^+^ *E. coli* was higher among conventionally raised cattle and their beef products, however, the number of cattle lots and ground beef samples with *clb*^+^ *E. coli* did not differ between CONV and RWA production systems. Therefore, this study does not indicate that use of antibiotics in beef production selects for *clb*^+^ *E. coli*. In fact, the types of antibiotic resistance genes identified within *clb*^+^ *E. coli* are among those frequently detected regardless of antimicrobial use^48,49^.

The whole genome sequencing of the *clb*^+^ *E. coli* in this study revealed several novel results regarding their phylogeny. Unlike human clinical samples^50,51^, frequency of *clb*^+^ is highest among phylogroup B1 *E. coli* isolated from beef. This may be a source-specific difference as animal *E. coli* strains often belong to group B1, whereas group B2 includes many human pathogenic strains^52–54^. There appears to be a preference for particular genetic backgrounds among the clades of *pks.* All CC95 *pks* sequences in this study as well as two reference bovine CC95 isolates KS-P003 and KS-P027, but none of the human clinical CC95 strains in clade 1, carried *fimH*15 suggesting host specificity among CC95 strains. Further, the potential mechanism of *pks* transfer appears to be specific to genetic background as well as *pks* sequence clades. Occasionally, however, the same mechanisms appear to allow *pks* transfer to different genetic backgrounds, as well as transfer of different *pks* sequence variants. Colibactin production may be independent of phylogeny of *clb* genes^55^, nevertheless, future studies are needed to assess the production and activity of Clb among cattle and meat-borne isolates.

In this study, 27% of sequenced isolates belonged to phylogroup B2, a phylogroup that possesses more virulence factors and increasingly has become dominant in industrialized nations^56,57^, where CRC is also on the rise^58^. Further, high association of colicin genes, *cdtB*, and *clb* among these isolates will likely contribute towards their success in gut colonization, leading to microbial dysbiosis and ultimate progression to CRC. However, the CC95 *pks* islands identified in this study cluster distantly from reference human CC95 strains, similar to a recently reported observation^50^. Therefore, for proper assessment of risk posed by cattle and meat-borne CC95 *pks*^+^ strains in human CRC, future studies aimed at establishing human gut colonization status and lesion formation in colonic tissues by these isolates are necessary.

It is well established how pathogenic *E. coli* such as *E. coli* O157:H7 contaminates beef during harvest and processing^59^, and herds of cattle with twenty percent fecal prevalence have greater than eighty percent prevalence on their hides^60^. Thus, *clb*^+^ *E. coli* may be present on hides and contaminated beef in a similar fashion as *E. coli* O157:H7. Over the last twenty years, hide and carcass directed interventions have been implemented to reduce *E. coli* O157:H7 contamination^18,61^. These interventions may have influenced *clb*^+^ *E. coli* on beef during this same time span. Historic data or samples are unfortunately lacking to address this. However, modern beef processing interventions focused on pathogen control may be what is confounding a more recent epidemiological report that cannot establish strong correlations between red meat consumption and CRC^62^. For instance, recent studies have shown an association of high red meat intake with an alkylating mutational signature in the colorectum, suggesting this to be a mechanistic link between CRC and red meat consumption^63^. Interestingly, *clb*^+^ *E. coli* also alkylates host DNA and lead to tumorigenic lesions^10^. One could speculate that the cohorts of individuals in the recent studies have been exposed to less *clb*^+^ *E. coli* (and other toxigenic *E. coli*) through the red meat they consume than those in the past.

Colorectal cancer is the third most common malignancy worldwide^1^ and is arguably associated with red meat consumption. Understanding the distribution and characteristics of *clb*^+^ *E. coli* in beef production and processing systems is the first step towards identifying microbial factors that may contribute to red meat associated CRC. Red meat, but not poultry and fish, is associated with increased risk of CRC. Therefore, expanding this study to the latter two, as well as other red meat sources is essential to draw conclusions regarding role of *clb*^+^ *E. coli* in red meat associated CRC.

## Methods

### Ethics statement

All *E. coli* isolates used in this work were archived strains from previous work^22–24^ where sets of arbitrarily selected *E. coli* were examined. Samples from cattle were collected at a commercial feedlot^22^ and immediately after commercial harvest processes at USDA–FSIS federally inspected establishments where humane animal handling was practiced^22–24^. All experimental protocols involving cattle were approved and carried out in accordance with relevant guidelines from USD-ARS-US Meat Animal Research Center, Institutional Biosafety Committee (IBC #2.1 and #3.0) and Institutional Animal Care & Use Committee (IACUC #81.0). This study is reported in accordance to ARRIVE guidelines.

### Study design

Generic *E. coli* isolates previously recovered and archived at the United States Meat Animal Research Center (USMARC) were utilized for the current study. First, generic *E. coli* (n = 1430) isolated from the colonic contents of cattle-at-harvest were utilized^22^. These samples were collected from 72 cattle lots (36 raised without antibiotics (RWA) and 36 raised conventionally (CONV) on a monthly basis (3 lots of each per month) over a period of one year. Colon contents were collected from colons immediately after carcass evisceration. Eviscera was tracked to coincide with targeted lots of cattle. Next, to examine *clb*^+^ *E. coli* across the beef production continuum, 232 generic *E. coli* isolated from three lots of fed beef cattle along different stages of beef processing continuum (feces and hides at feedlot, feces and hides at abattoir, pre-evisceration carcasses, final carcasses, and packaged strip loins) were utilized^23^. Finally, for finished beef products, 1074 generic *E. coli* collected from 507 retail ground beef samples (267 with RWA label claims and 240 without, considered CONV) obtained from 6 U.S. cities were examined^24^. The prevalence of *clb*^+^ *E. coli* was unknown, therefore the full collections of strains from each study were examined in lieu of a power analysis.

### *clb* screening assay

To screen *E. coli* isolates for the *pks* island, a multiplex PCR targeting *clb* genes at the 5’ and 3’ regions of the 54 kb *pks* island from *E. coil* Nissel 1917 (GenBank accession No: CP022686) was designed and utilized. An aliquot of the overnight culture growing at 37 °C in Luria Bertani (LB) broth (Fisher Scientific, Pittsburg, PA) was combined with BAX lysis buffer (Hygiena, Camarillo, CA) following manufacturer’s recommendations to generate template DNA. Primer sequences are provided in Supplementary Table T2. Amplicons were resolved on 1.2% agarose gels and stained with ethidium bromide for visualization.

### Antimicrobial susceptibility screening assay

Forty-four *clb*^+^ *E. coli* (35 colon isolates + 9 ground beef isolates) were evaluated for susceptibility towards commonly used meat processing interventions. These isolates were arbitrarily chosen to represent diverse cattle production systems, months, and regions. Isolate *E. coli* O157:H7 FSIS-4 from the USMARC collection, which was previously characterized for its susceptibility towards the antimicrobial treatments^25^, was utilized as a reference. The following antimicrobial solutions were prepared in tap water at room temperature: 2% lactic acid (LA, pH = 2.3) (Sigma-Aldrich, St. Louis, MO), 200 ppm peroxyacetic acid (PAA, pH = 3.4) (BLITZ organic, FMC Corp., Philadelphia, PA), and 300 ppm 1,3-dibromo-5,5-dimethyl hydrantoin (BR, pH = 7.0) (Albemarle, Baton Rouge, LA). For non-treated control, an equal volume of sterile, deionized water was used. PAA and BR concentrations were determined by following manufacturer’s recommendation using a PAA test kit (Peroxychem, Philadelphia, PA) and bromine photometer II (Hanna Instrument, Smithfield, RI) respectively.

Individual isolates were grown overnight in LB broth at 37 °C. Optical density at 600 nm (OD600) of each culture was adjusted to an equivalent cell density of ~ 1.0X10^8^ CFU/mL. Each individual culture (200 μL) was mixed with 800 μL of each antimicrobial solution in deep-well 96 well blocks (USAScientific, Ocala, FL). Lastly, a non-chemical treatment using hot water (80 °C) was examined, where 200 μL culture was mixed with pre-warmed 800 μL sterile deionized water in thin-walled polypropylene tubes (USAScientific, Ocala, FL) and incubated in 80 °C water-bath (PolyScience, Niles, IL). Treatment durations were 5 min for LA or hot water, and 30 s for PAA or BR. At the end of each exposure time, 100 μL of treated cells were transferred to 900 μL Difco D/E neutralization broth (BD), held at room temperature for 1 h, then 100 μL was transferred to 900 μL LB broth and incubated at 37 °C for 6 h. At the end of the incubation period, fold reductions in OD600 of treated samples compared to untreated samples were calculated from three independent experiments and compared to *E. coli* O157:H7 FSIS-4.

### Fresh beef inoculation study

Beef inoculation studies were performed as described previously with slight modifications^44^. Four *clb*^+^ *E. coli* (Col222, Col317, Col1011, and Gb644) were selected based on their phenotype on Sorbitol MacConkey agar containing 5-bromo-4-chloro-3-indolyl-β-D-glucuronide (BCIG-SMAC; Oxoid Basingstoke UK), which allowed their differential enumeration from *E. coli* O157:H7 (*E. coli* O157:H7, straw yellow color and *clb*^+^ *E. coli* purple to violet color). Each *clb*^+^ *E. coli* isolate and *E. coli* O157:H7 FSIS-4 were individually grown for 16 h at 37 °C in LB broth and OD600 was adjusted to a cell density of ~ 10^8^ CFUmL. An inoculum was prepared by mixing equal volumes of each culture with final cell density adjusted to ~ 10^7^ CFU/mL. For each intervention, two beef flanks were used, and surface pH measured before treatment (mean pH 5.6 to 5.8). The exterior of each flank was marked to 16 sections of 25 cm^2^ using branding ink and inoculated with ~ 10^5^ CFU/25 cm^2^ as described previously^44^, then freshly prepared antimicrobial compounds (2% LA, 200 ppm PAA, or 300 ppm BR) were applied individually using a handheld electrostatic spray gun (ES sprayer Model VP200ESK, Victory Innovations Co., St. Louis Park, MN) without the electrostatic mode from a distance of 15 cm, at 110 lb/in^2^ for 1 min with a flow rate of 0.32 L/min. After each treatment, excess liquid was allowed to drip for 30 s. In the case of heat treatment, vacuum packaged flanks were pre-warmed to 31 °C in a water bath to avoid heat loss during the treatment. Dry steam was generated using Vapamore MR-100 steamer (Vapamore, Scottsdale, Az) and applied to each 25 cm^2^ section for 15 s. During heat treatment, meat surface temperature was continuously monitored to reach approximately 80–82 °C using Digi-Sense thermometer with type J thermocouple (Cole-Parmer, Vernon Hills, IL). After each treatment, the 25 cm^2^ sections were randomly cut and individually put into sterile filtered bags, resuspended in 75 mL of Dey-Engle broth (BD) supplemented with 200 mM K2HPO_4_, homogenized, and tenfold serially diluted as previously described^44^ Appropriate dilutions were spiral plated onto BCIG-SMAC agar, then incubated at 37 °C for 24 h for enumeration, and CFUs were counted. Bacterial counts were transformed to log CFU/cm^2^ and reduction in Log CFU/ cm^2^ of treated samples compared to untreated samples was calculated.

### Whole genome sequencing of *pks*^+^*E. coli* isolates

For whole genome analysis, 69 *clb*^+^ *E. coli* isolates (55 colon, 12 ground beef, and 2 continuum isolates) were selected based on cattle types, months, and regions to have the greatest variety of possible sources represented. Genomic DNA (gDNA) was extracted from overnight culture (LB broth, 37 °C) and purified using Qiamp DNA mini kit (Qiagen, Valencia, CA). gDNA was sheared to an average size of 350 bp using Covaris microtubes (Covaris Inc, Woburn MA). Illumina sequencing libraries were prepared using a TruSeq DNA PCR free LP kit (Illumina, San Diego, CA). Paired read sequencing was performed on MiSeq platform using kit v2 (300 cycles) (Illumina, San Diego, CA).

### Genome assembly, analysis, and annotation

An in-house genome analysis pipeline was used to assemble the genomes, screen them against Center for Genomic Epidemiology (CGE) databases and obtain the assembly statistics. Detail of the in-house genome assembly pipeline is provided in Supplementary Data File SD2. Genome assembly quality was further assessed with QUAST^64^. Fifteen genomes were discarded due to low coverage and/or poor sequence quality. Metadata for the remaining fifty-four *clb*^+^ *E. coli* isolates (41 colon, 11 ground beef, and 2 continuum isolates) are provided in Supplementary Data File SD1. Phylogroups were predicted with ClermonTyping 21.03. The *pks* island was identified through high sensitivity mapping of new genomes against the reference *pks* sequence from *E. coli* strain IHE3034 (GenBank Accession Number: AM229678.1) using Geneious mapper in Geneious prime 2020.1.2. Draft shovill genomes were annotated with NCBI prokaryotic genome annotation pipeline^65^.

### Phylogenetic analysis

For phylogenetic analysis of newly identified *pks* islands, ~ 51 kb region spanning the first amino acid of first gene *clbA* to the last amino acid of last gene *clbS* was extracted from 38 individual contigs, each harboring the full length *pks* island. Regions upstream of *clbR* containing variable number of tandem repeat (5’-ACAGATAC-3’) were removed before analysis^55^. These *pks* islands were mapped against each-other and 10 reference *pks* sequences from GenBank (Supplementary Table T3) using MAFFT aligner^66^ in Geneious prime V2020.1.2 (Biomatters). The best fit model for maximum parsimony phylogenetic analysis was determined to be GTR using Akaike’s information criteria (AIC) by Modeltest-NG v0.1.7^67,68^.

For phylogenetic analysis of core genomes of *clb*^+^ isolates, *E. coli* LY180 (Genbank Accession number: CP006584.1) was selected as the closest matching reference genome using Patric 3.6.10^69^. Prophage sequences within the reference genome were identified with PHASTER^70,71^ and masked using Bedtools^72^. Core genomes of 54 *clb*^+^ isolates and the reference genome were aligned using ParSNP version 1.2. The best fit model for maximum parsimony phylogenetic analysis was determined to be GTR + I + G4 using AIC by Modeltest-NG v0.1.7^67,68^.

Maximum likelihood phylogenetic tree was constructed using iQ-tree1.6.10, and visualized with iTol v6.

### Statistical analyses

Statistical analysis for *clb* prevalence was performed using Fisher’s Chi-squared test (GraphPad Prism). For flank inoculation assay, log reductions in CFU/cm^2^ of *clb* isolates and *E. coli* O157:H7 reference isolate after treatment were compared using one-way analysis of variance (ANOVA).

## Supplementary Information

Supplementary Information 1.

Supplementary Information 2.

## Data Availability

All genome sequences have been uploaded to NCBI under Bioproject no. PRJNA761561. GenBank accession numbers for individual PGAP annotated genomes are provided in Supplementary Data File SD1.
