# Supplementary material for: Characterization of Escherichia coli harboring colibactin genes (clb) isolated from beef production and processing systems
Source: Sci Rep. 2022 Mar 29;12:5305. doi: 10.1038/s41598-022-09274-x (PMC8964808; doi:10.1038/s41598-022-09274-x)
Supplement: Supplementary file 2 — Supplementary Information 2. [file 41598_2022_9274_MOESM2_ESM.pdf]

Characterization of *Escherichia coli* Harboring Colibactin Genes (*clb*) Isolated from Beef Production and Processing Systems

Manita Guragain[1], John W. Schmidt[1], Norasak Kalchayanand[1], Aaron M. Dickey[1], and Joseph M Bosilevac[1]*

[1]US Department of Agriculture, Agricultural Research Service, U.S. Meat Animal Research Center, P.O. Box 166, State Spur 18D, Clay Center, Nebraska 68933

*Author for correspondence. Tel: 402-763-4225; Fax: 402-762-4149; E-mail: mick.bosilevac@usda.gov

Genome assembly, analysis, and annotation. Genomes were assembled using the Shovill version 1.0.4 pipeline (available at https://github.com/tseemann/shovill). Shovill incorporated the following steps: 1.) genome size and sequencing coverage estimations were performed using Mash version 2.2 [1]; 2.) Illumina adaptor sequences were removed using Trimmomatic version 0.39 [2]; 3.) Lighter version 1.1.2 was used to correct sequencing read errors [3]; 4.) FLASH version 1.2.11 was used to merge and extend short reads [4]; 5.) reads were assembled using SPAdes version 3.14.0 [5]; and 6.) corrected minor assembly errors by mapping reads back to contigs, removed short contigs, low coverage contigs, and homopolymers using BWA version 0.7.17-r1188 [6], SAMtools version 1.10 [7] and Pilon version 1.23 [8]. SerotypeFinder (version 2018_09_24) was used to determine *E. coli* serotypes [9]. Multi-locus sequence types (MLST) were determined using the program “MLST” (version 2019_05_08) [10]. Antimicrobial resistance genes and chromosomal mutations known to confer antimicrobial resistance were identified using ResFinder version 2020_02_06 [11-13]. Previously described virulence factors were identified using VirulenceFinder version 2.0 [14,15]. Local implementations of the Center for Genomic Epidemiology (CGE) “finder” tools utilized Blast+ version 2.10.0 [13] to query CGE databases updated on 07/2021. GC, N50, and L50 statistics were obtained using the stats script from BBMap version 38.79.

Supplementary Table T1: Prevalence of *clb*$^{+}$ *E. coli* across beef-processing continuum

Sample matrix	Number of generic *E. coli* isolates (*clb*$^{+}$ *E. coli*)			
	Lot#1	Lot#2	Lot#3	Total
Feedlot hide sponge (FH)	12	12	12	36
Feedlot fecal swab (FF)	12 (1)[a]	12	12	36 (1) [a]
Harvest hide sponge (HH)	12	12 (1) [a]	12	36 (1) [a]
Harvest fecal swab (HF)	12	12	12	36
Pre-intervention carcass (PC)	12	12	12	36
Final carcass (FC)	12	4	12	28
Strip loin (SL)	12	12	0	24
Total	84 (1) [a]	76 (1) [a]	72	232

[a] Number in parentheses represents number of *clb*$^{+}$ *E. coli*.

Supplementary Table T2: Primer sequences used for *clb* screening based on *clb* genes from *E. coli* Nissel 1917.

Primers	Sequence	Reference
clbA forward	5’-CTAGATTATCCGTGGCGATTC-3’	[16]
clbA reverse	5’-CAGATACACAGATACCATTCA-3’	[16]
clbQ forward	5’-CTTGTATAGTTACACAACTATTTC-3’	[16]
clbQ reverse	5’-TTATCCTGTTAGCTTTCGTTC-3’	[16]

Supplementary Table T3: GenBank Accession number of reference *pks* islands

Strain	Origin	GenBank accession number
Escherichia. coli SI-NP020	Bovine	AP024130.1
Escherichia. coli KS-P019	Bovine	AP024123.1
Escherichia. coli UPEC129	Human	CP071522.1
Escherichia. coli JML285	Human	AP024114.1
Escherichia. coli IHE3034	Human	AM229678.1
Escherichia. coli ABU83972	Human	CP001671
Escherichia. coli ECSC054	Human	AP024112.1
Citrobacter koseri ATCC BAA-895	Human	CP000822.1
Enterobacter aerogenes EA1509E	Human	FO203355.1
Klebsiella pneumoniae subsp. pneumoniae 1084	Human	CP003785.1
Escherichia. coli KS-P003	Bovine	OM103703*
Escherichia. coli KS-P027	Bovine	OM103704*

*; Full length sequences for analysis were kindly provided by Dr. Frederick Auvray and will be available at the respective accession numbers in NCBI.

REFERNCES

1 Ondov, B. D. *et al.* Mash: fast genome and metagenome distance estimation using MinHash. *Genome Biology* **17**, 132, doi:10.1186/s13059-016-0997-x (2016).

2 Bolger, A. M., Lohse, M. & Usadel, B. Trimmomatic: a flexible trimmer for Illumina sequence data. *Bioinformatics* **30**, 2114-2120, doi:10.1093/bioinformatics/btu170 (2014).

3 Song, L., Florea, L. & Langmead, B. Lighter: fast and memory-efficient sequencing error correction without counting. *Genome Biology* **15**, 509, doi:10.1186/s13059-014-0509-9 (2014).

4 Magoč, T. & Salzberg, S. L. FLASH: fast length adjustment of short reads to improve genome assemblies. *Bioinformatics* **27**, 2957-2963, doi:10.1093/bioinformatics/btr507 (2011).

5 Bankevich, A. *et al.* SPAdes: a new genome assembly algorithm and its applications to single-cell sequencing. *J Comput Biol* **19**, 455-477, doi:10.1089/cmb.2012.0021 (2012).

6 Li, H. & Durbin, R. Fast and accurate short read alignment with Burrows–Wheeler transform. *Bioinformatics* **25**, 1754-1760, doi:10.1093/bioinformatics/btp324 (2009).

7 Li, H. *et al.* The Sequence Alignment/Map format and SAMtools. *Bioinformatics* **25**, 2078-2079, doi:10.1093/bioinformatics/btp352 (2009).

8 Walker, B. J. *et al.* Pilon: an integrated tool for comprehensive microbial variant detection and genome assembly improvement. *Plos One* **9**, e112963, doi:10.1371/journal.pone.0112963 (2014).

9 Joensen, K. G., Tetzschner, A. M., Iguchi, A., Aarestrup, F. M. & Scheutz, F. Rapid and Easy In Silico Serotyping of *Escherichia coli* Isolates by Use of Whole-Genome Sequencing Data. *J Clin Microbiol* **53**, 2410-2426, doi:10.1128/JCM.00008-15 (2015).

10 Larsen, M. V. *et al.* Multilocus sequence typing of total-genome-sequenced bacteria. *J Clin Microbiol* **50**, 1355-1361, doi:10.1128/JCM.06094-11 (2012).

11 Zankari, E. *et al.* PointFinder: a novel web tool for WGS-based detection of antimicrobial resistance associated with chromosomal point mutations in bacterial pathogens. *J Antimicrob Chemother* **72**, 2764-2768, doi:10.1093/jac/dkx217 (2017).

12 Bortolaia, V. *et al.* ResFinder 4.0 for predictions of phenotypes from genotypes. *J Antimicrob Chemother* **75**, 3491-3500, doi:10.1093/jac/dkaa345 (2020).

13 Camacho, C. *et al.* BLAST+: architecture and applications. *BMC Bioinformatics* **10**, 421, doi:10.1186/1471-2105-10-421 (2009).

14 Joensen, K. G. *et al.* Real-time whole-genome sequencing for routine typing, surveillance, and outbreak detection of verotoxigenic *Escherichia coli. J Clin Microbiol* **52**, 1501-1510, doi:10.1128/JCM.03617-13 (2014).

15 Malberg Tetzschner, A. M., Johnson, J. R., Johnston, B. D., Lund, O. & Scheutz, F. In Silico Genotyping of *Escherichia coli* Isolates for Extraintestinal Virulence Genes by Use of Whole-Genome Sequencing Data. *J Clin Microbiol* **58**, doi:10.1128/JCM.01269-20 (2020).

16 Morgan, R. N., Saleh, S. E., Farrag, H. A. & Aboulwafa, M. M. Prevalence and pathologic effects of colibactin and cytotoxic necrotizing factor-1 (Cnf 1) in

Escherichia coli: experimental and bioinformatics analyses. *Gut Pathog* **11**, 22, doi:10.1186/s13099-019-0304-y (2019).

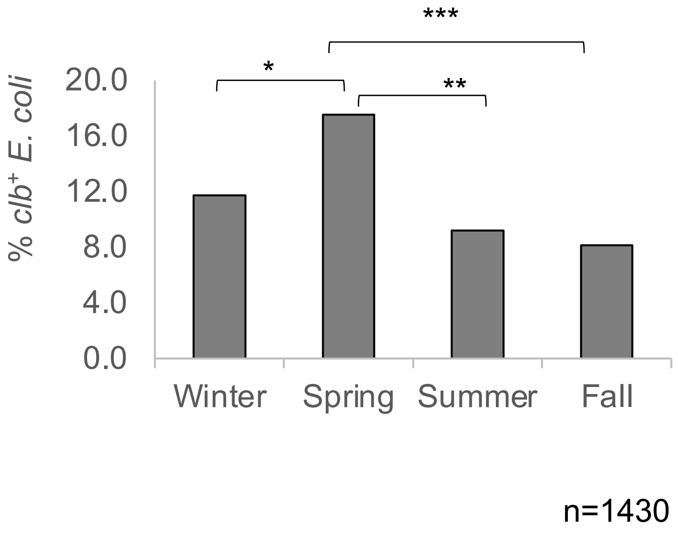

Supplementary Figure S1. Percentage prevalence of *clb*$^+$ *E. coli* in cattle across the seasons. Statistical significance was calculated by Fisher's exact test. *, significant (p<0.05); ***, extremely significant (, p<0.001).

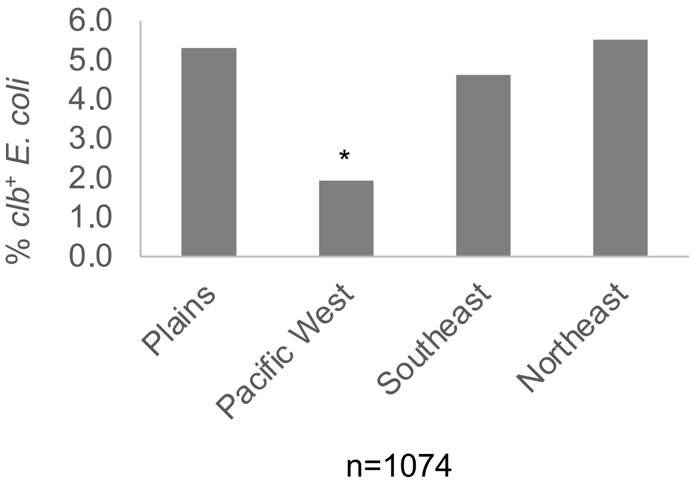

Supplementary Figure S2. Percentage prevalence of *clb*$^{+}$ *E. coli* in ground beef among generic *E. coli* by geographical regions. Statistical significance was calculated by Fisher's exact test . *, significant ($p<0.05$); **, very significant (, $p<0.005$).

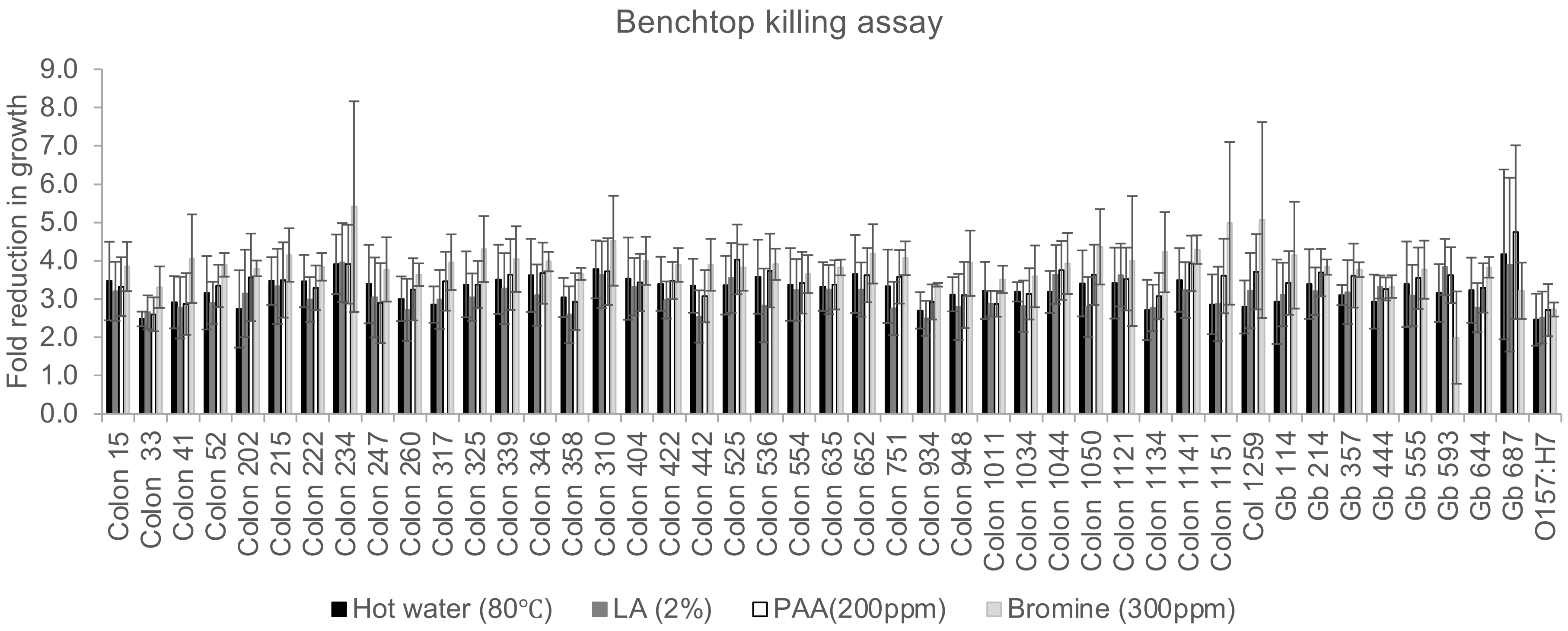

Supplementary Figure S3. Fold reduction in growth of *clb*⁺ *E. coli* upon treatment with antimicrobials. Data represents average of three independent biological experiments.

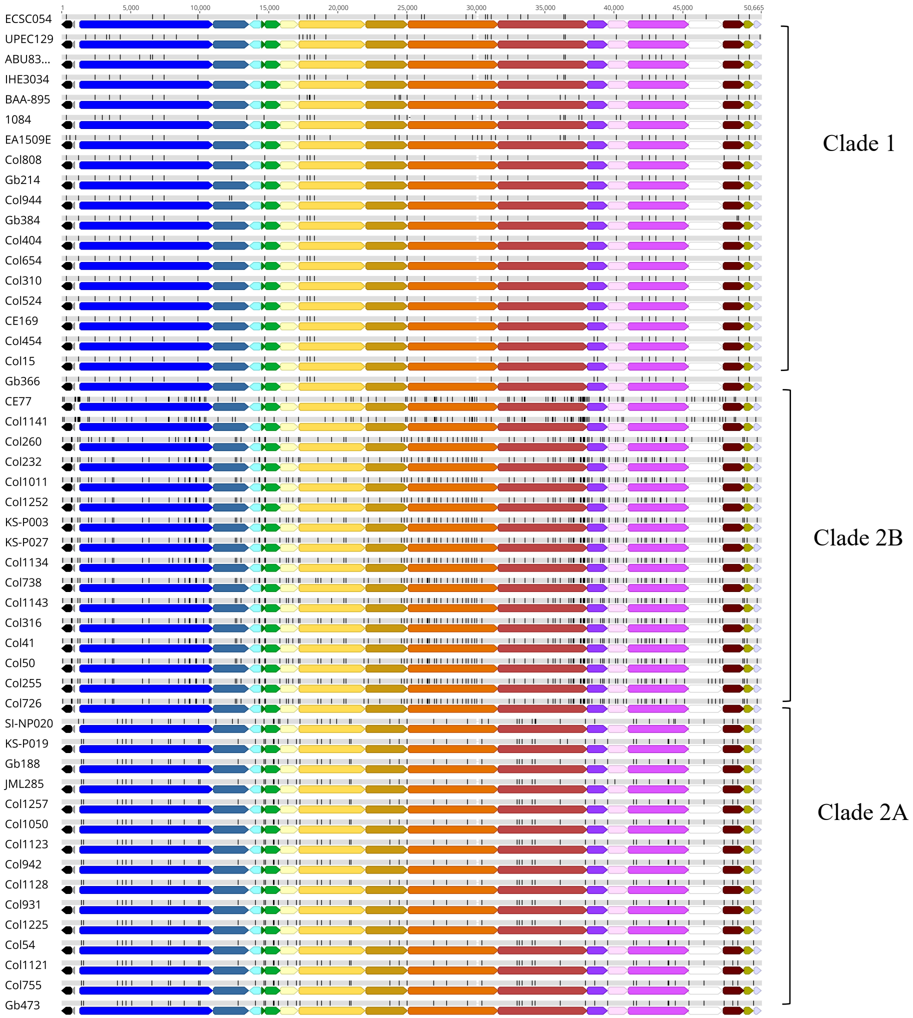

Supplementary Figure S4. Nucleotide sequence alignment of newly identified *pks* islands together with reference sequences using MAFFT in Geneious prime. clbA, clbR, ClbB, clbC, clbD, clbE, clBF, clbG, clbH, clbI, clbJ, clbK, clbL, clbM, clbN, clbO, clbP, clbQ, clbS genes.
